# Treat Iron-Related Childhood-Onset Neurodegeneration (TIRCON)—An International Network on Care and Research for Patients With Neurodegeneration With Brain Iron Accumulation (NBIA)

**DOI:** 10.3389/fneur.2021.642228

**Published:** 2021-02-22

**Authors:** Ivan Karin, Boriana Büchner, Florence Gauzy, Angelika Klucken, Thomas Klopstock

**Affiliations:** ^1^Department of Neurology, Friedrich-Baur-Institute, University Hospital of the Ludwig-Maximilians-University (LMU), Munich, Germany; ^2^Office of International Science Cooperation, Bavarian Research Alliance (BayFOR), Munich, Germany; ^3^Hoffnungsbaum e.V., Velbert, Germany; ^4^Munich Cluster for Systems Neurology (SyNergy), Munich, Germany; ^5^German Center for Neurodegenerative Diseases (DZNE), Munich, Germany

**Keywords:** NBIA, TIRCON, patient registry, clinical network, orphan disease, movement disorder

## Abstract

In order to improve clinical care, coordinate research activities and raise awareness for the ultra-orphan Neurodegeneration with Brain Iron Accumulation (NBIA) disorders, a group of NBIA clinicians and researchers, industry partners and patient advocacies from six European countries, Canada and the US joined forces in 2010 to set-up the collaborative initiative TIRCON (Treat Iron-Related Childhood-Onset Neurodegeneration). As a research project, TIRCON received funding in the 7th Framework Programme (FP7) of the European Union (EU) from 2011 to 2015. After successful and timely completion of the initial FP7 project, funding and donations from industry and patient organizations have sustained the further development of TIRCON's dedicated clinical research infrastructure and its governance architecture, as well as the ongoing efforts undertaken in the NBIA community to establish a network of care. From the beginning, the University Hospital of the Ludwig-Maximilians-University in Munich, Germany has been coordinating the TIRCON initiative. It consists of 8 work packages, of which the first double-blind, placebo-controlled, randomized, multi-site clinical trial in NBIA (deferiprone in PKAN, completed) and a global patient registry and biobank, currently comprising baseline and follow-up data of > 400 NBIA patients have gained particular importance. Here we describe TIRCON's history with all the challenges and achievements in diagnosing and treating NBIA. Today, TIRCON lays the ground for future clinical care and research. In these times, it may also serve as a good example of well-directed governmental funding and fruitful international scientific collaboration.

## Introduction

Over the last decade, interest in rare diseases has steadily grown both politically and scientifically. To raise awareness, EURORDIS, a European non-governmental organization for rare diseases funded mainly by patient organizations (www.eurordis.org), the European Commission (EC) and private corporations, initiated the first Rare Disease Day® in February 2008. Ever since, rare diseases have gained increased coverage within popular media.

Per definition, a condition is considered a rare (or orphan) disease if its prevalence is <5:10,000. Compared to widespread diseases such as cerebral stroke or ischemic heart disease, this number may sound negligible: however, all 5,000–8,000 rare diseases together affect no <27 million patients alone in the European Union (EU) (1). Even among orphan diseases, Neurodegeneration with Brain Iron Accumulation (NBIA) disorders are still exceedingly rare and are thus referred to as ultra-orphan diseases. For instance, the estimated incidence of one of the more frequent NBIA forms (Pantothenate Kinase-Associated Neurodegeneration, PKAN), is around 2 in 1,000,000 live births among the non-African world population (2). The causative gene, *PANK2*, was the first NBIA gene discovered, published in 2001 by the Hayflick group (3). To date, mutations in 10 genes have been linked to NBIA (4). NBIA disorders show a broad phenotypic spectrum ranging from movement disorders manifesting as dystonia, spasticity, and parkinsonism to other predominantly neurological symptoms such as optic atrophy, neuropsychiatric symptoms, and cognitive decline. NBIA disorders can be distinguished from other diseases by the eponymous MRI patterns of increased brain iron levels (5).

**Figure 1:**
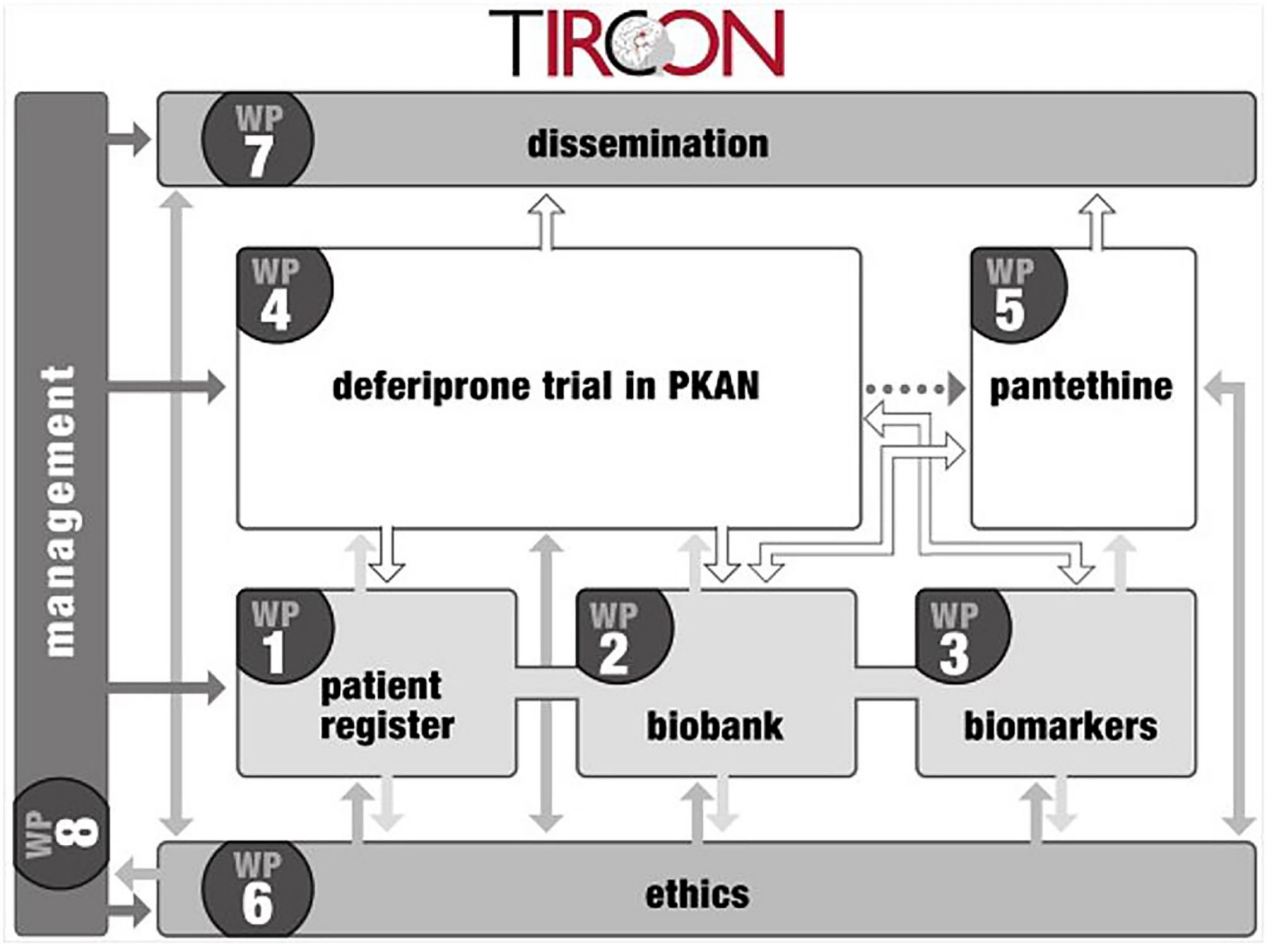
Overview of TIRCON work packages.

The TIRCON project received EU funding from the 7th Framework Programme (FP7) with 5.2 million € from Nov 1st, 2011 to Oct 31st, 2015. It consists of 8 different work packages, with the international NBIA patient registry and biobank (work package 1) being the nucleus for further clinical and basic research (see Figure 1) (6). TIRCON is coordinated by the Friedrich-Baur-Institute at the Department of Neurology of the University Hospital of Munich supported by the Bavarian Research Alliance (BayFOR).

## Impact on Clinical Research: the Patient Registry and Its Structure

**Figure 2:**
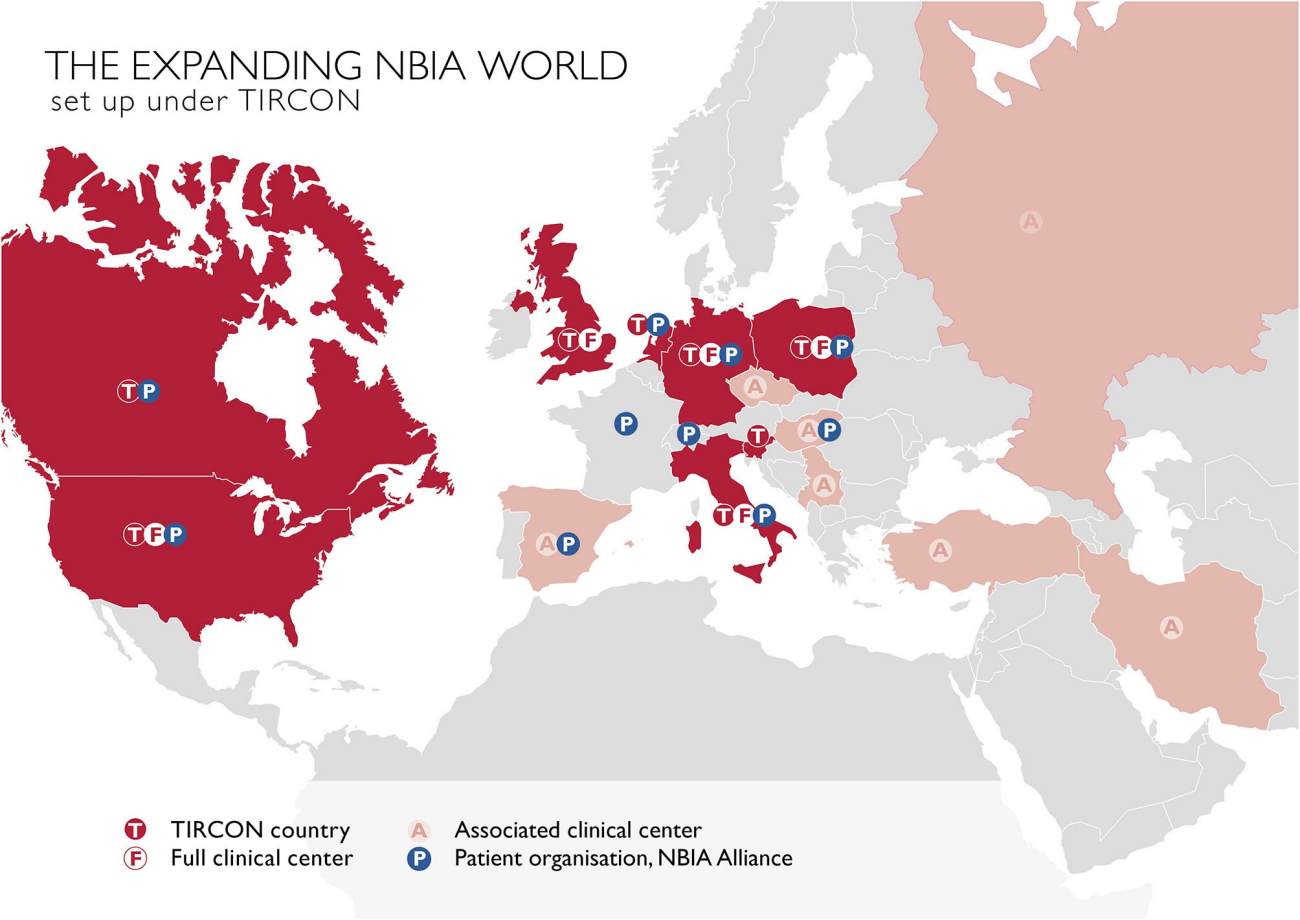
TIRCON centers: T / F, initially involved TIRCON partners, full clinical centers and their respective countries; A, centers/countries, joined the network after the project start; P, patient organizations.

The main goal of TIRCON's work package 1 has been to create the first international NBIA patient registry. The NBIA patient registry has been fully operational since 2013. Since the end of the FP7 funding, TIRCON has received further monetary support from the international NBIA patient organizations gathered under the umbrella of the NBIA Alliance and from pharmaceutical companies including ApoPharma Inc. (Toronto, Canada), CoA Therapeutics (San Francisco, CA; USA) and Retrophin Inc. (San Diego, CA; USA) To date, clinical centers in several European countries, the United States and Asia are contributing to the registry, including the TIRCON core partners who laid the foundation for the establishment of the registry as well as clinical centers which have been associated over time (see Figure 2). All clinical centers have been approved by their respective local ethics committee to include patients into the patient registry.

**Figure 3:**
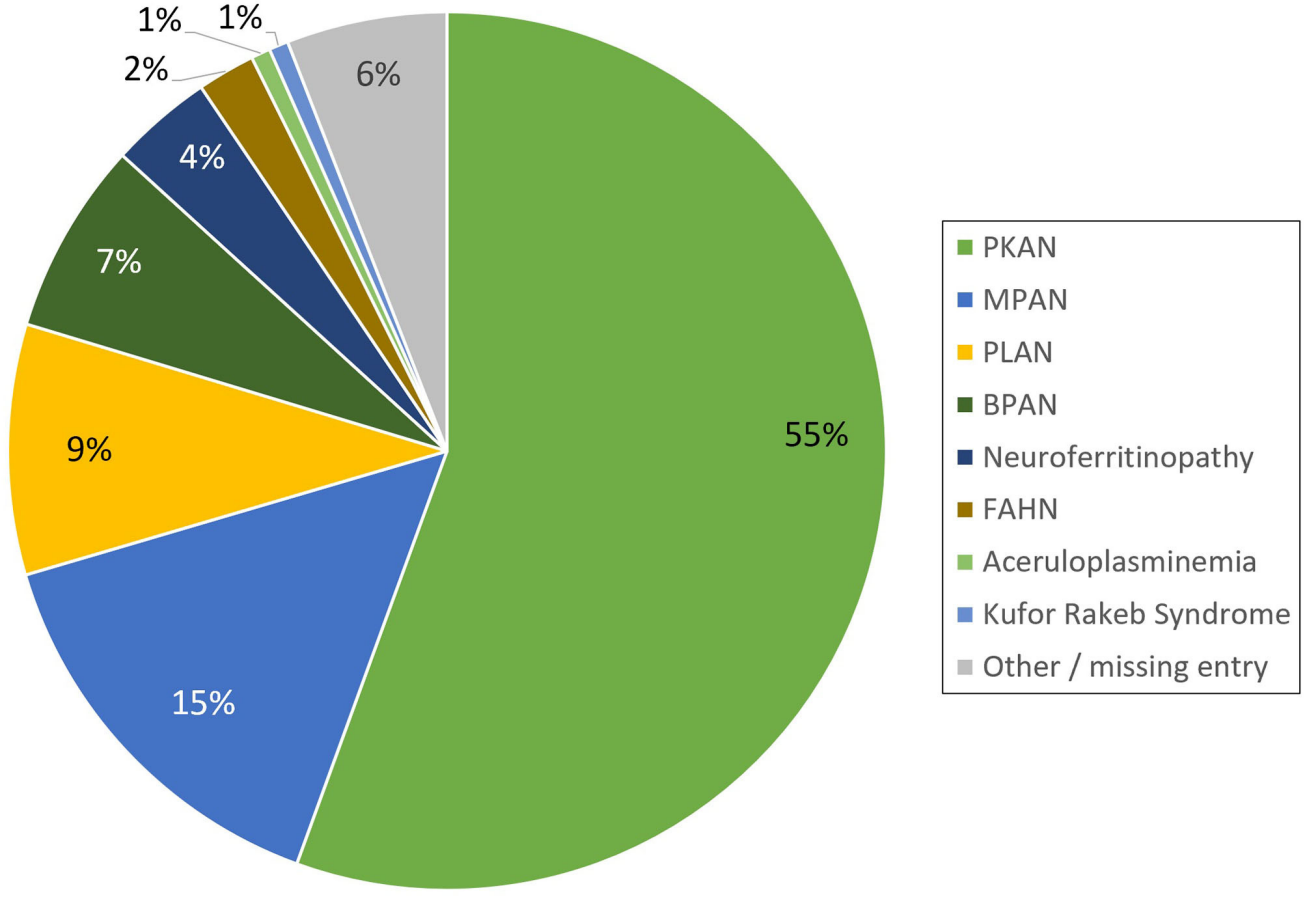
Distribution of NBIA subtypes in the TIRCON patient registry (PKAN, Pantothenate Kinase-Associated Neurodegeneration; MPAN, Mitochondrial-membrane Protein-Associated Neurodegeneration; PLAN, *PLA2G6*-Associated Neurodegeneration; BPAN, Beta-propeller Protein-Associated Neurodegeneration; FAHN, Fatty Acid Hydroxylase-Associated Neurodegeneration).

**Figure 4:**
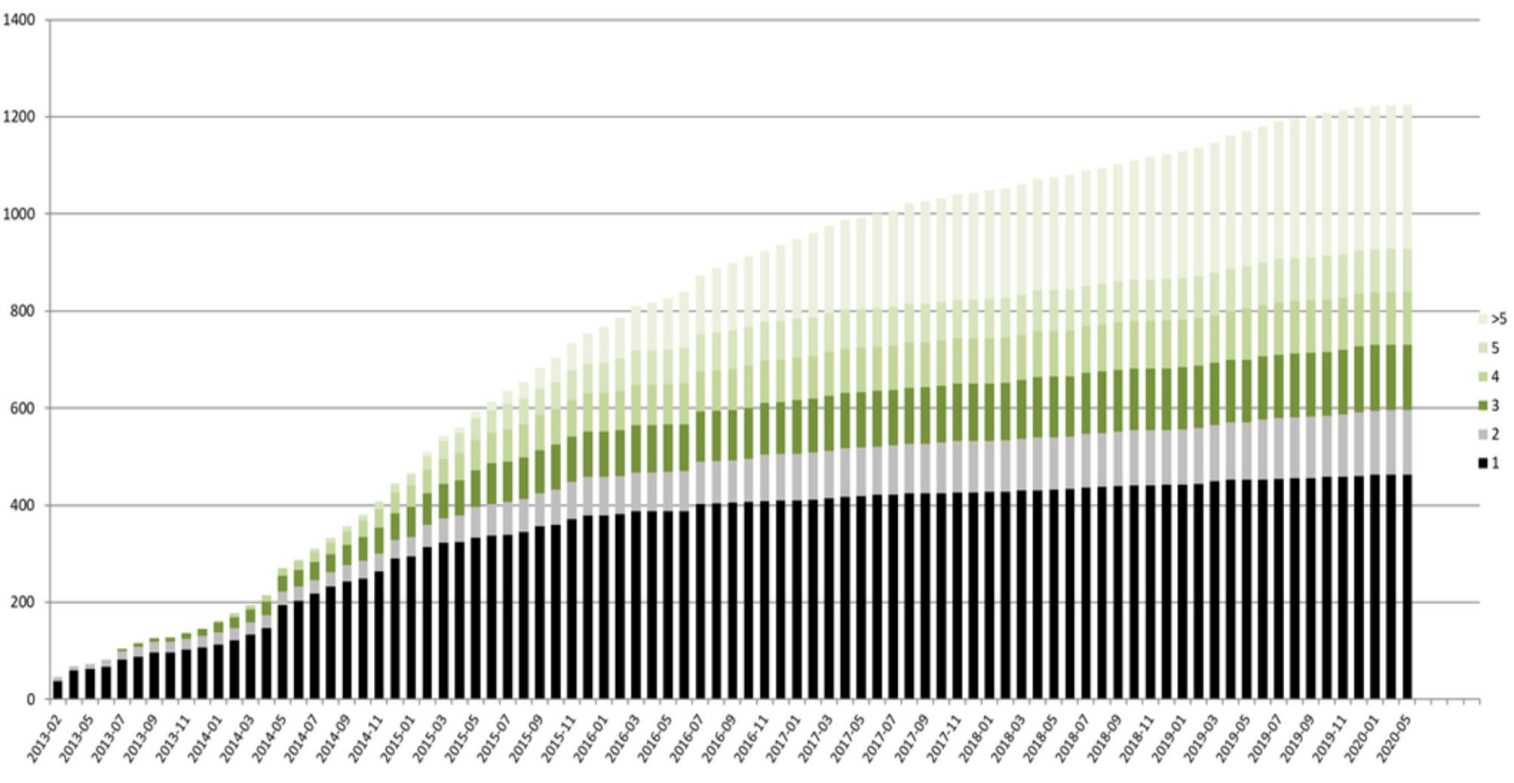
TIRCON registry—baseline (black) and follow-up visits (different colors used, depending on the total number of follow-up visits) over time.

The registry is open for all patients with a genetically established diagnosis of NBIA or clinically suspected NBIA. It is designed as a multicenter, prospective, cross-sectional, and longitudinal study with yearly follow-up visits. The first patient was enrolled in February 2013. Since then, > 420 patients with different NBIA subtypes have been recruited (see Figure 3). Including follow-up visits, the registry contains > 1,200 entries (see Figure 4).

Before inclusion, patients or their caregivers sign an appropriate informed consent in their respective local language. All patients undergo a complete body examination and a detailed neurological examination by a neurologist trained in movement disorders, and have their medical history taken at each visit. In order to standardize symptom reporting, each investigator is asked to provide clinical data in accordance with the Human Phenotype Ontology (HPO) (7). Recorded demographic data include year of birth, gender, ethnic background and country of residence of the patient, as well as ethnic background, country of residence and potential consanguinity of the parents. The date of onset of first symptoms is recorded as well as the date of clinical diagnosis. Whenever possible, genetic results are obtained. If a patient presents without genetic testing, next generation sequencing (NGS) or whole exome sequencing (WES) can be initiated at most full clinical centers. All pharmacological and non-pharmacological therapies such as physical therapy or special dietary regimes are recorded, including start date, end date (if applicable) and dosage. The patients are asked to bring all relevant medical records with them including neuroimaging and discharge letters. If consented, a family tree can be generated linking diseased family members to each other. Results from routinely performed blood assessments (i.e., blood count, liver enzymes, creatinine, iron, ferritin, creatine kinase, ceruloplasmin, lactate, TSH) can be collected, too. To assess progression of motor symptoms, two well-established scales have been implemented. The Barry-Albright Dystonia (BAD) Scale measures the severity of dystonia in eight body regions (eyes, mouth, neck, trunk, and each upper and lower extremity) and ranges from 0 (no dystonia) to 32 (severe dystonia) (8). To rate the severity and progression of motor symptoms not limited to dystonia, parts I, II, III, and VI of the Unified Parkinson's Disease Rating Scale (UPDRS) are obtained (9). Although validated for patients with Parkinson's disease, the UPDRS seems to reflect impairment of motor functions even in patients without pronounced Parkinsonian features sufficiently (clinical experience from treating physicians). Quality of sleep is recorded through the Pittsburgh Sleep Quality Index (PSQI) (10). To assess quality of life, each patient is requested to fill out the Pediatric Quality of Life (PedsQL™) Inventory version 4.0; in addition, for patients up to the age of 25 years a separate scale for the caregiver is provided (11).

The data is entered into a secured online platform. Its security and privacy concept builds upon the security architecture of the German mitoNET registry and is conformant with the German Data Protection Act (12). Every entry is monitored by staff from the Friedrich-Baur-Institute. Monitored entries are then reimbursed at predefined rates depending on the integrity of the data. Clinical data may be shared upon request in accordance with all ethical and data protection requirements.

While collecting more and more data throughout the years, we hope to gain better insight into natural disease progression. Growing patient numbers enrolled into the registry will help to increase trial readiness by enabling the formation of large cohorts.

## Impact on Clinical Research: Clinical Trials

Thanks to intense efforts by medical professionals and especially patient organizations in recruiting patients for the patient registry, the first two phase III trials in NBIA could be conducted. The randomized, double-blind, controlled trial of deferiprone for PKAN enrolled 88 patients for phase III and its open-label extension study (13). Initially designed as an investigator-initiated trial, ApoPharma Inc. (Toronto, Canada; now part of Chiesi Group) soon took over as the primary sponsor. Deferiprone has shown to be a well-tolerated and safe drug in PKAN. As proof of concept, it decreased brain iron levels in the basal ganglia significantly and seemed to slow disease progression but did not reach overall significance. After completion of the deferiprone trial, a second phase III trial was conducted to evaluate the safety and effectiveness of fosmetpantotenate for the treatment of PKAN (14). The study was sponsored by Retrophin Inc. (San Diego, CA; USA). The study failed to reach its primary and secondary endpoints and the open-label extension trial was discontinued (15).

Before TIRCON, no randomized trials evaluating disease-modifying treatment options were available but only case reports or small open-label studies (16, 17).

## Impact on Patient Care: Clinical Expertise

With TIRCON, several NBIA clinical centers have been founded that now serve as centers of excellence where neurologists and neuropediatricians can refer patients to in order to confirm an NBIA diagnosis or to get a second opinion on standard of care. For patients with this ultra-rare disease with which even most specialists are unfamiliar, it is crucial to know that they have a center where they can turn to in order to receive a consultation. As a result of their targeted networking and collaboration, NBIA specialists published the first consensus guidelines for the treatment of an NBIA disorder (18), a work that was supported by TIRCON.

## Impact on Basic Research: NBIA Biobank

In close association with the NBIA patient registry, TIRCON's work package 2 has focused on establishing an international NBIA biobank. At each patient visit, either for the registry and/or for the deferiprone trial, patients and their caregivers are asked to provide biosamples for the biobank. Each individual patient (or his/her caregiver) consents by signing a separate informed consent form. Each sample kit contains two EDTA tubes for plasma and DNA analysis, one PAXgene™ tube for RNA analysis and one tube for a urine sample. All samples are initially stored at −20 or −80°C at the local center before being sent along with basic anonymized demographic and medical history data to the Technical University of Munich (TUM) for central storage. The biosample collection is used in TIRCON's work package 3 for genomic, proteomic, transcriptomic and metabolomic analyses to identify biomarkers reflecting disease course and treatment effects. The biobank is permitted to share its biosamples with external collaborators. Recently, a paper on residual PANK2 activity in patients' erythrocytes has been published by a group of non-TIRCON researchers who received biosamples from the biobank (19).

Members of the TIRCON consortium have contributed significantly to the discovery of several new NBIA genes such as *c19orf12, WDR45*, and *COASY* (20–22).

## Impact on Basic Research: Discovering New Compounds

It was shown that in Drosophila models the enzymatic defect in PKAN can be bypassed by downstream compounds such as pantethine (23). In TIRCON's work package 5, two TIRCON partners, Acies Bio (Ljubljana, Slovenia) and the University Medical Center Groningen (UMCG, Netherlands), worked together to conduct basic research on the efficacy and safety of pantethine and pantethine derivatives in cell and mouse models. The fruitful collaboration resulted in a successfully filed patent to the European Medicines Agency (EMA) (24). A clinical trial with a very similar approach using 4'-phosphopantetheine is currently recruiting patients in North America (25).

## Pivotal Work in the Background: Ethics, Dissemination, and Management

The activities in work packages 6–8 were mostly related to non-scientific aspects of conducting a large-scale research project as TIRCON.

Work package 6 focused on ethical, regulatory and legal aspects concerning access to and use of patient data and biomaterials, as well as the involvement of animals in preclinical research. The preparation, translation and adaptation to local or national requirements of informed consent forms for the international registry, biobank and the multi-site deferiprone trial were also part of this work package. Child-friendly ICFs were made available. Preparing for the clinical trial in Munich and the different sites abroad included missionary work with local academic and public health institutions. The safety during the clinical trial was monitored by an appointed Data and Safety Monitoring Board (DSMB). Clear intellectual property rules were needed and implemented to guarantee transparent and scalable cooperation among the two industry partners as well as between the academic and the industry partners.

Work package 7 raised awareness for NBIA by running the official TIRCON web page, handling press relations and releasing educational material for both the public and the scientific community and put emphasis on educational training (See also below). In both work packages, the architecture designed to support the registry, the biobank and the clinical research has kept its value and validity.

The activities in work package 8 included overall administrative and financial coordination. A specific challenge was to facilitate and manage the full participation of Canadian and US-partners receiving funding from the EU. This work package monitored in addition good practice in communication. Here again, the governance mechanisms put in place for TIRCON in the Consortium Agreement are still valid today.

## Tircon From the Perspective of Patient Organizations

**Figure 5:**
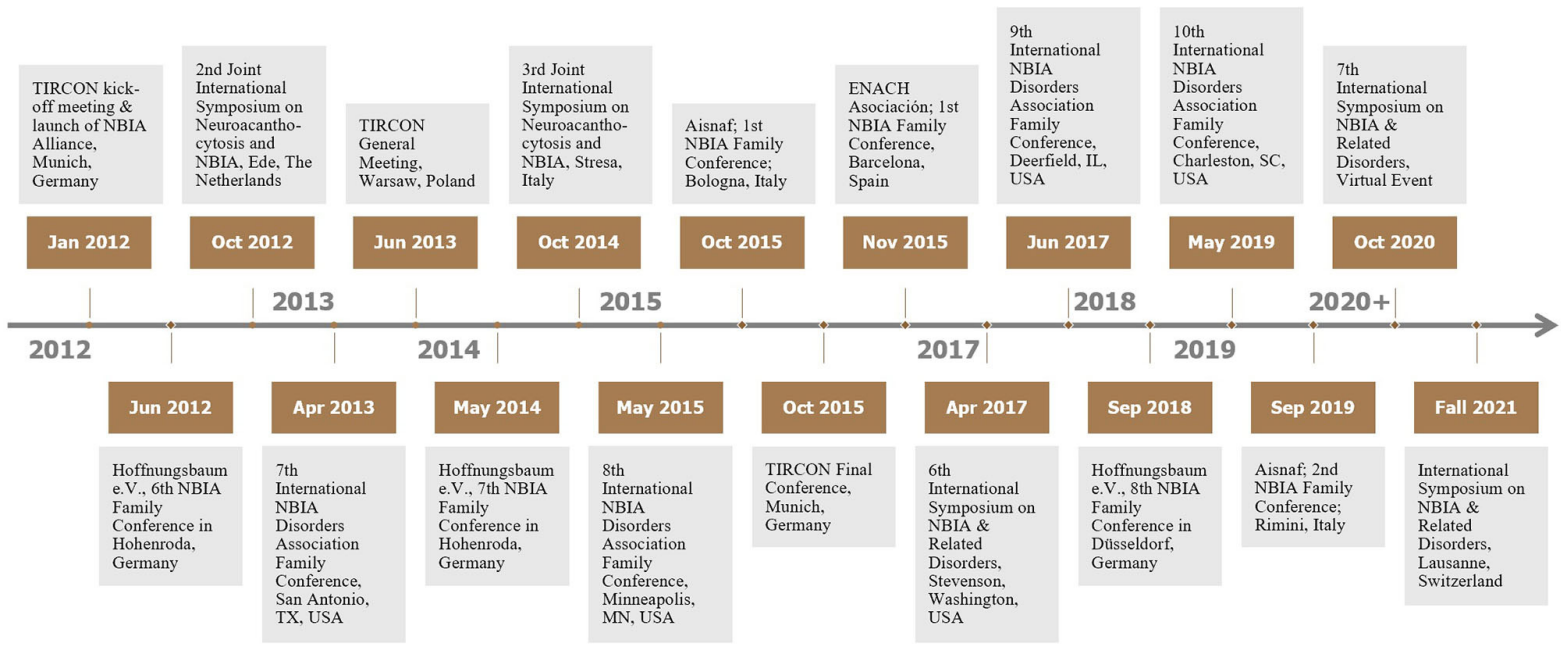
Major NBIA events since 2012—TIRCON meetings, scientific symposia, family conferences.

The NBIA Disorders Association (NBIADA, USA) and Hoffnungsbaum e.V. (HoBa, Germany) have been involved in TIRCON from the very beginning of the FP7 application process. Finally, both became full partners in TIRCON, including budget granted by the EU for their responsibilities in work package 7. HoBa, due to being located in an EU member state, took over the work package lead and thus became member of the Scientific Steering Committee with its monthly meetings, represented in absence by the NBIA Disorders Association. This way, patient organizations have been actively integrated in all aspects and decisions of this consortium throughout the project. This framework enables a trust-based cooperation between the patient representatives and the other partners within TIRCON. HoBa and NBIADA have developed dissemination instruments, including brochures, presentations, newsletter-articles and web-based information that are tailored to patient families and target groups like clinicians, scientists or further advocacies. Moreover, scientific NBIA symposia, TIRCON meetings, and NBIA family conferences have been held in TIRCON partner countries to create networking occasions for a mutual exchange among all interested stakeholders in the field of NBIA on research projects in TIRCON and best practices in clinical care for NBIA (see Figure 5).

Regarding Europe, TIRCON has significantly contributed to the setup or empowerment, respectively, of the clinical NBIA expert centers in Munich, Milan, Warsaw and Newcastle, establishing them as first-rate contact points for patients and clinicians from all over the world requiring consultation with NBIA experts. As a consequence, TIRCON has eased the work of patient organizations remarkably. They now can connect the patient families directly with clinical NBIA experts. It was far more difficult before TIRCON for Patient Advocacy Organizations (PAOs) and patients to identify and reach out to clinical NBIA experts. While in the USA at least one NBIA center in Portland, Oregon had already been established since the 1990's, there was not a comparable infrastructure for NBIA patients in Europe before TIRCON.

A first step to strengthen the patient community was the launch of the “NBIA Alliance” as part of TIRCON dissemination tasks at the TIRCON kick-off meeting in Munich in 2012. The NBIA Alliance, which is not a legal entity but a federation of independent patient associations, was founded by HoBa, NBIADA, the Italian patient advocacy Aisnaf and the French association AIDNAI (www.nbiaalliance.org). In the following years, TIRCON has supported the emergence of new NBIA patient organizations according to the subsidiarity principle. Subsequently, new NBIA associations were founded in Spain (ENACH Asociación), the Netherlands (Stichting Ijzersterk), Switzerland (NBIA Suisse), Canada (NBIA Canada), Poland (NBIA Polska), and Hungary (NBIA Hungary). Interestingly, in countries where active new NBIA patient organizations could be established, clinical NBIA expert centers emerged or were empowered by the respective NBIA advocacy. NBIA research activities are strongly supported or even initiated by the new patient advocacies, often preferably in their home countries. This again demonstrates how the deliberate empowerment of the patient organizations in TIRCON up to becoming co-responsible partners has been instrumental to success.

The PAOs were partners in work package 1 (Patient registry), 4 (Deferiprone trial for PKAN patients), and 6 (Ethics), where they contributed patients' needs and points of view and have continuously supported patient recruitment for registry to date. Challenges the small associations had to face as TIRCON partners included the bureaucratic demands within such an EU-funded project and the high proportion of work that had to be done on a volunteer basis. However, the PAOs consider that the impact TIRCON has had since then on the NBIA patient community and the development of their advocacies made all efforts worthwhile.

## Discussion

TIRCON has set up a solid foundation for future NBIA research by bringing together this formerly scattered NBIA community of basic scientists and clinicians, while including patient organizations from the very beginning. Even several years after completion of the FP7 project, the NBIA community has not dispersed but remains highly collaborative: the patient registry and biobank are fully operational and have secured financial support for the years to come, sustainable collaborations in basic and clinical science have developed and academic experts, industry partners and patient representatives meet on a regular basis to network. Recently the NBIA Disorders Association, the patient advocacy in the US, hosted the 7th International Symposium on NBIA & Related Disorders. The event took place for the first time as a virtual conference due to the Covid-19 pandemic from September 30th to October 3th 2020. TIRCON has contributed to publish > 30 peer-reviewed papers including some key publications in discovering new NBIA genes (21, 22). What defines and differentiates TIRCON is the close integration and the invaluable commitment of the patient organizations. The number of patient organizations has grown ever since, with new members in North America and Europe (see above).

Still, several challenges remain in the future. For severely affected patients, a face-to-face consultation may not be a feasible option due to their critical condition. Besides telephone or e-mail, other means of communication are necessary in such cases. The concept of ‘flying-doctors’ with the physician coming to the patient and not the other way round seems promising, but financial reimbursement and time consumption constitute limiting factors. The Covid-19 pandemic has shown that online consultations *via* video chats may offer an alternative. Fortunately, legal hurdles for online consultations have been reduced over the last few years. When conducting clinical trials in ultra-rare diseases, not only is recruiting a sufficient number of patients challenging but also defining reasonable endpoints. Further basic research is urgently needed to discover relevant biomarkers of disease progression. The lack of disease-specific scales is another limiting shortcoming. Thus, to assess all relevant symptoms of the disease, more than one rating scale must be applied, with each one of these scales, besides not being validated for NBIA, only reflecting some aspects of the disease. In PKAN however, first steps have been taken in creating disease-specific scales rating motor function and activities of daily living (26, 27). The TIRCON clinical center in Poland is currently validating a disease-specific rating scale for MPAN (private communication).

In conclusion, TIRCON can serve as a model for an international public and private funded research project creating more than the required deliverables: truly enduring bonds and new ways of working together in science, industry and patient care. Beyond the novelty of the TIRCON's research architecture, it remains true that such initiative can only prosper on the ground of personal dedication from professionals, patients and their families.

## Author Contributions

IK wrote the first draft of the manuscript. BB, FG, AK, and TK contributed substantially to the manuscript as well as providing critical comments to the content. All authors approved the submitted version.

## Conflict of Interest

TK served as coordinating investigator of the FORT trial; received research funding from Retrophin Inc.; served as coordinating investigator of the deferiprone in PKAN randomized and extension trial; received research funding from ApoPharma Inc.; received support from the European Commission 7th Framework Programme (FP7/2007-2013, HEALTH-F2-2011, grant agreement No. 277984, TIRCON) and from the European Reference Network for Rare Neurological Diseases (ERN-RND), co-funded by the European Commission (ERN-RND: 3HP 767231); provided consulting services to CoA Therapeutics and TM3 Therapeutics; received travel support from ApoPharma Inc. BB provided consulting services to Retrophin Inc. and ApoPharma Inc. IK received travel support from ApoPharma Inc. FG received support from the European Commission 7th Framework Programme (FP7/2007-2013, HEALTH-F2-2011, grant agreement No.277984, TIRCON). AK received support from the European Commission 7th Framework Programme (FP7/2007-2013, HEALTH-F2-2011, grant agreement No. 277984, TIRCON); received travel support from Retrophin Inc.
